# Alterations in Muscle Coordination to Reduce Knee Joint Loading for People with Limb Loss

**DOI:** 10.1007/s10439-025-03682-6

**Published:** 2025-01-24

**Authors:** Jiayu Hu, Ziyun Ding, Anthony M. J. Bull

**Affiliations:** 1 School of Engineering, University of Birmingham, Birmingham, B15 2TT UK; 2 Department of Bioengineering, Imperial College London, London, UK

**Keywords:** Amputee, Musculoskeletal modelling, Knee joint moment, Knee contact force, Muscle coordination, Kinematics

## Abstract

**Purpose:**

People with unilateral transtibial amputation experienced a higher incidence of knee osteoarthritis due to abnormal movement patterns. This study aimed to explore alternations in muscle coordination to reduce mechanical burdens in their daily activities.

**Methods:**

Five males with unilateral transtibial amputation were recruited. Two daily activities (level walking and standing up from a chair) were simulated using muscle-driven simulation. Two cost functions were applied: minimising the knee adduction moment and minimising both the knee adduction moment and knee flexion moment. This enabled the identification of altered muscle coordination and any possible changes in kinematic patterns.

**Results:**

For people with unilateral transtibial amputation, reducing knee adduction angles during stance was found effective in reducing knee adduction moments. To mitigate peak contact forces at the intact knee, muscle activations of vasti and gastrocnemius should be decreased, while muscle activations of soleus should be increased during activities of level walking and standing up from a chair.

**Conclusion:**

Our study suggested that minimising knee adduction moments was effective in reducing joint loading during level walking while minimising both knee adduction moments and knee flexion moments was effective during standing up from a chair. Therefore, the alteration of muscle coordination should be carefully designed for different daily activities.

**Supplementary Information:**

The online version contains supplementary material available at 10.1007/s10439-025-03682-6.

## Introduction

Amputation is a life-changing disability. Approximately 1.2 million people experience significant limb amputations each year due to vascular diseases, trauma and cancer on a global scale [1]. Moreover, the increasing incidence of diabetes and an ageing population are expected to lead to an increase in the number of amputations. It is anticipated that the amputee population will double by 2050 [2].

People with lower limb loss commonly exhibit abnormal movement patterns. A notable example is the presence of asymmetric gait, as evidenced by the higher knee flexion of the intact limb compared to the amputated limb in the early stance of level walking [3, 4]. To counter the loss, they may also adopt new muscle coordination strategies to restore movement similarity to pre-amputation conditions [5]. Studies have shown increased muscle activation in knee flexors at the intact limb during walking [6]. In addition, greater activation of the hip extensor has been observed [7]. Greater muscle activation, as a consequence, leads to increased joint loading at the intact knee and hip joints, indicating an elevated risk of osteoarthritis (OA) [8–10]. This increased risk is linked to a higher incidence of OA in individuals with limb loss [11].

Gait retraining, such as the alternation of foot progression angle [12, 13], lateral trunk lean [14] and medial thrust [15], has been developed to alleviate symptoms or slow the progression of OA. This is achieved through a modification of the gait kinematic pattern, thereby reducing the knee adduction moment, a crucial indicator for the medial knee contact force distribution. Reducing the excessive medial knee contact force is pivotal in non-surgical treatment of knee OA [16–18]. By reducing the knee adduction moment, the force distribution across the knee is rebalanced, resulting in a reduced force at the medial knee compartment [17, 19]. It has also been reported that a reduction in the combined knee adduction moment and knee flexion moment could be a more effective means of reducing medial knee contact force [20].

In the presence of pathological gait due to OA, altering muscle coordination strategies is also crucial for achieving the therapeutic goal of reducing joint loading. For people with knee OA, excessive knee loading can be mitigated by adopting a "gastrocnemius avoidance" gait [21]. In theory, the internal knee contact force can be manipulated to several times bodyweight without changing kinematics [22, 23]. Musculoskeletal simulation provides a practical platform to identify the effect of altered muscle coordination strategies on joint loading [21–26]. This is achieved by customising a new cost function to explore other potential muscle activation patterns, considering the muscle redundancy around the knee [24, 25]. However, for people with limb loss, it remains unclear how these gait retraining strategies might impact the remaining muscle activation and whether the altered muscle coordination can effectively reduce joint loading on the non-affected side.

The main objective of this study was to explore new muscle activation patterns aimed at mitigating excessive knee joint loading at the intact limb for people with unilateral lower limb loss. The musculoskeletal simulation was employed to investigate two daily activities: level walking and standing up from a chair. Alterations in muscle coordination were identified by using two cost functions: minimising the knee adduction moment or minimising both the knee adduction and flexion moments. A secondary objective was to identify the modified kinematic pattern when the knee adduction and flexion moments were minimised, in order to inform gait retraining for people with unilateral lower limb loss.

## Materials and Methods

### Participants and Motion Data

Motion data were collected from five males with unilateral transtibial amputation (age: 34 ± 2 years; weight: 86 ± 9 kg; height: 180 ± 3 cm). They were eligible for the study as they had been using their definitive prostheses for at least six months and were able to walk independently without walking aids for twelve minutes. The detailed experimental protocol is described in Ding et al [8], and in brief here.

Marker trajectories were measured using a 10-camera optical motion capture system (100 Hz, Vicon, Oxford, UK); ground reaction forces were measured using force plates (1000 Hz, Kistler Type 9286B; Kistler Instruments ltd, Winterthur, Switzerland). Following the static standing trials, participants were asked to perform the level walking with a self-selected speed and standing up from a chair with each foot placed in the centre of each force plate. Each task was repeated three times in compliance with all relevant instructions. All procedures were approved by the Imperial College Research Ethics Committee (Reference 16IC3562) and the NHS Research Ethics Committee (REC reference 16/LO/1715). Informed consent of each participant was received before the experiment began.

### Musculoskeletal Model of Transtibial Amputation

A musculoskeletal model of an individual with a unilateral transtibial amputation was created by modifying a full-body musculoskeletal model [27]. This full-body model comprises 14 articulating rigid bodies, totalling 21 degrees of freedom. Specifically, six degrees of freedom were assigned to the pelvis and the ground, three to the lumbar joint, and six to each leg: three in the hip joint, two in the knee joint and one in the ankle joint. Notably, for the original knee joint with a single flexion/extension degree of freedom, an additional abduction/adduction degree of freedom was added to allow the rotational motion in the frontal plane [28]. Additionally, this model comprises 80 Hill-type muscle-tendon units [29]. Each muscle unit was defined as massless actuators with active and passive components (series and parallel) of force generation [30]. Several modifications were made following Wilson [27] to simulate a transtibial amputation at the midpoint of the tibia and fibula. First, half of the shank was removed, resulting in a 50% reduction of the mass of the residual shank and a proximal shift of its centre of mass to 25% of the distance between the knee and ankle joints. Subsequently, the ankle joint and eleven muscle-tendon units across the ankle joint were removed. A generic prosthesis model, consisting of a socket, pylon and foot, was created, where the socket and pylon were rigidly connected with equal lengths [27]. The prosthetic ankle was modelled as a pin joint with one degree of freedom, enabling plantarflexion and dorsiflexion [31, 32].

The musculoskeletal model was first scaled to match each participant using the marker trajectories from the static standing trial [33]. Scaling factors were calculated by comparing the distances between the experimental markers on the participants and the corresponding model markers. Then, the scale tool scaled the body segment dimensions and inertial properties, as well as the optimal fibre length and tendon slack length of muscle-tendon actuators, according to the scaling factor [33]. After scaling, the inverse kinematics tool calculated the generalised joint coordinates while minimising errors between the experimental marker trajectories during dynamic trials (level walking and standing up from a chair) and the corresponding model marker trajectories. Additionally, the residual reduction algorithm tool was utilised to resolve dynamics inconsistencies [33]. This tool refined generalised joint coordinates and model mass properties to minimise errors between the model dynamics and experimental ground reaction forces.

### Simulations of Level Walking and Standing Up from a Chair

**Fig. 1:**
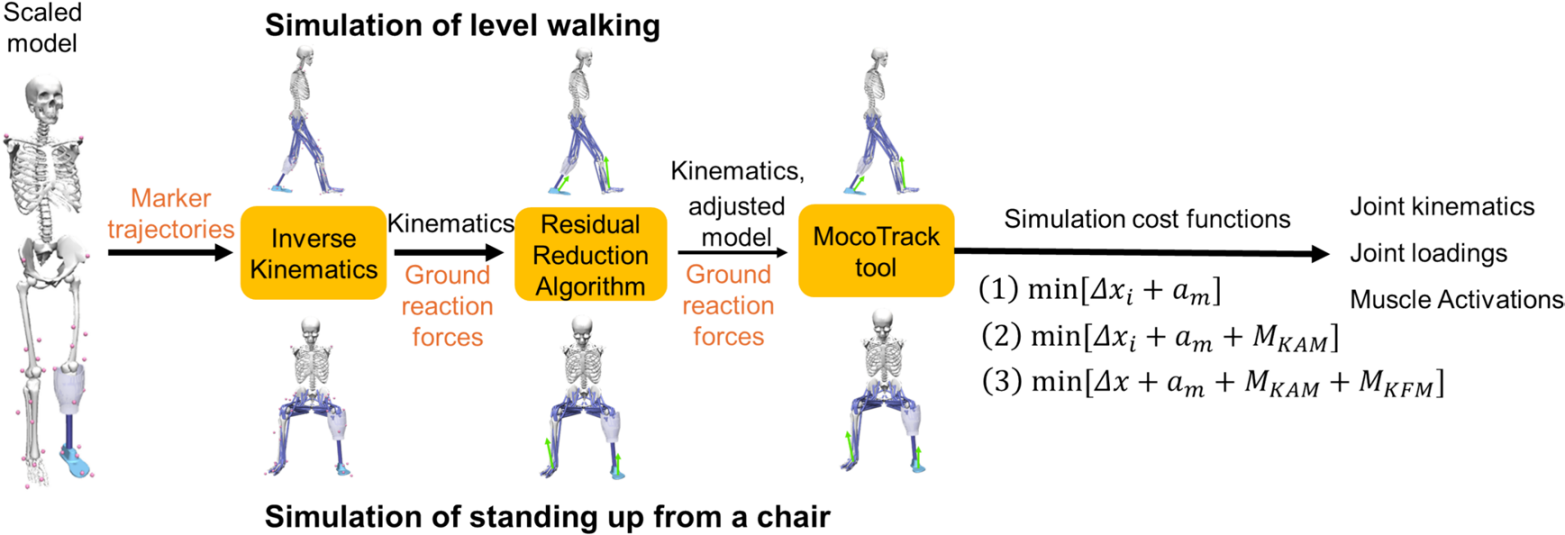
Simulation workflow to investigate the muscle coordination strategies using three cost functions. The process begins with using the collected marker trajectories (orange colour) and a scaled model to calculate joint kinematics (black colour) via the inverse kinematics (IK) tool. Next, the collected ground reaction forces (orange colour) and computed kinematics were input into the residual reduction algorithm (RRA) to address dynamic inconsistencies, producing adjusted joint kinematics and model (black colour). Finally, these adjusted results, along with the ground reaction forces, are fed into the OpenSim MocoTrack module to perform muscle-driven simulations of level walking, tracking experimental motion capture data to calculate joint kinematics, joint loading, and muscle activations (black colour).

Muscle-driven simulations of level walking and standing up from a chair were implemented using the MocoTrack tool in the OpenSim Moco (musculoskeletal optimal control) software toolkit [34] (as shown in Fig. 1). MocoTrack enables the use of customised cost functions, in addition, enables the identification of modified kinematics when the customised cost functions are satisfied [35]. We used the following three cost functions at the intact limb:

$\text{min}[\Delta {x}_{i}+{a}_{m}]$(1) : by minimising (i) the tracking error and (ii) muscle effort, as a default cost function to generate a baseline simulation [34].

1 $$\min \mathop \smallint \limits_{{t_{0} }}^{{t_{f} }} \left( {w_{1} \mathop \sum \limits_{i = 1}^{I} w_{i} \left( {x_{i} \left( t \right) - u_{i} \left( t \right)} \right)^{2} + w_{2} \mathop \sum \limits_{m = 1}^{M} a_{m}^{2} \left( t \right)} \right)dt$$

where, ${t}_{0}$ represents the initial time and ${t}_{f}$ the ending time for the simulation; ${w}_{1}$ and ${w}_{2}$ are the weight factors for the first and second terms, respectively; ${w}_{i}$ is the weight factor for each deviation squared between the experimental joint coordinates (${x}_{i}$) and the simulated joint coordinates (${u}_{i}$), with $I$ equals to 21, representing the total joint degrees of freedom of the musculoskeletal model. $M$ equals to 90, representing the total 69 remaining muscles plus 21 residual and reserve actuator controls; ${a}_{m}$ denotes the muscle effort.

$\text{min}[\Delta {x}_{i}+{a}_{m}+{M}_{KAM}]$(2): by minimising (i) the tracking error; (ii) muscle effort and (iii) knee adduction moment (${M}_{KAM}$) as:

2 $$\min \mathop \smallint \limits_{{t_{0} }}^{{t_{f} }} \left( {w_{1} \mathop \sum \limits_{i = 1}^{I} w_{i} \left( {x_{i} \left( t \right) - u_{i} \left( t \right)} \right)^{2} + w_{2} \mathop \sum \limits_{m = 1}^{M} a_{m}^{2} \left( t \right) + w_{3} M_{KAM}^{2} \left( t \right)} \right)dt$$

where, ${w}_{3}$ represents the weight factor for the third term.

$\text{min}[\Delta x+{a}_{m}+{M}_{KAM}+{M}_{KFM}]$(3) : by minimising (i) the tracking error, (ii) muscle effort, (iii) knee adduction moment (${M}_{KAM}$), and (iv) knee flexion moment (${M}_{KFM}$) as:

3 $$\min \mathop \smallint \limits_{{t_{0} }}^{{t_{f} }} \left( {w_{1} \mathop \sum \limits_{i = 1}^{I} w_{i} \left( {x_{i} \left( t \right) - u_{i} \left( t \right)} \right)^{2} + w_{2} \mathop \sum \limits_{m = 1}^{M} a_{m}^{2} \left( t \right) + w_{3} M_{KAM}^{2} \left( t \right) + w_{4} M_{KFM}^{2} \left( t \right)} \right)dt$$

where, ${w}_{4}$ is the weight factor for the fourth term.

During simulations, weight factors of ${w}_{1}$ and ${w}_{2}$ were set to 0.11 and 0.16, respectively, following previous research [34]. ${w}_{\text{i}}$ values were manually adjusted to minimise the errors between the experimental and simulated joint coordinates. The values for ${w}_{3}$ and ${w}_{4}$, both set to 0.11, were determined through iterative reduction to minimise solver time, while ensuring that knee joint loading remained unchanged despite variations in the values [34]. After solving the tracking problems, predictive quantities, including the knee adduction moment, knee flexion moment, and total knee and hip contact forces, were extracted using *MocoTrajectory*, which retains solutions during dynamic optimisation [34].

### Data Analysis and Statistics

For each participant, three trials of walking and standing up from a chair were simulated. In level walking, the simulation commenced at the heel strikes and concluded at the toe-off for both the amputated and intact limbs. As for standing up from a chair, the simulation commenced at the seat-off as identified by the zero force on the chair's force plate, and concluded at the state of standing still, as identified based on the velocity of markers at the pelvis (no greater than 0.1 m/s) [36, 37]. Simulated trials were normalised within a 0-100% range to represent a cycle. Muscle activations, joint kinematics and joint loading were then averaged across three trials for each of the three cost functions at the intact limb: (1) default, (2) minimising the knee adduction moment and (3) minimising the knee adduction moment and knee flexion moment. For comparison, muscle activations, joint kinematics, and joint loading at the amputated limb were also extracted using the default cost function.

Moco Toolkit cannot directly compute contact forces for the medial or lateral compartments of the knee joint. Therefore, we utilised the following validated linear regression to quantify medial compartment contact force [38]

4 $$F_{med} = a + b \times M_{KAM} + c \times M_{KFM}$$

where $a$, $b$ and $c$ are regression parameters, and their values are dependent on the activities of walking and standing up from a chair, as $a=0.85$, $b=37$ and $c=-12$ for walking, and $a=0.53$, $b=22$ and $c=9$ for standing up from a chair, respectively.

Statistical analyses were performed using SPSS (IBM SPSS Statistics 26, Chicago, USA). The normality of the data was first assessed through a Shapiro-Wilk Test. A two-sided paired t-test was applied for comparing normally distributed data, while a two-sided Wilcoxon signed-rank test was applied for non-normally distributed data. Simulated knee adduction moment, knee flexion moment, knee contact force and hip contact force from cost functions (2) and (3) were compared with those predicted from cost function (1). The square of the Pearson correlation coefficient (R^2^) was calculated between the experimental and simulated joint kinematics. In addition, the correlation between the percentage change of knee contact force (i.e., total knee contact force and the medial knee contact force) and the percentage change of knee adduction moment or knee flexion moment were assessed using *R*^2^ for both activities. Correlation strength was categorised as good (*R*^2^ ≥ 0.75), moderate (0.50 < *R*^2^ < 0.75) or poor *R*^2^ ≤ 0.50, respectively [38]. The significance level was set at 0.05 for all statistical analyses

## Results

Simulated joint kinematics exhibited moderate to good correlation with the corresponding experimental data across all participants (0.640 ≤ *R*^2^ ≤ 0.998), as shown in SI Fig. 1 and S1 Table 1 in the supplementary file. The largest error was found in the knee abduction/adduction angles of the intact limb during standing up from a chair, with an average error of 4.7°.

**Table 1:** Simulated peak knee flexion moments and knee adduction moments in the intact limb using three cost functions: (1) default, (2) minimising the knee adduction moment (MinKAM) and (3) minimising the knee adduction moment and knee flexion moment (MinKAM&KFM) during level walking and standing up from a chair

	Level walking			Standing up from a chair		
	Default	MinKAM	MinKAM&KFM	Default	MinKAM	MinKAM&KFM
Knee Flexion moments (% BW × HT)						
First peak	1.93 (0.36)	**1.50 (0.32)**	**1.50 (0.30)**	9.58 (2.68)	**7.97 (2.11)**	**7.80 (2.02)**
Second peak	1.29 (0.21)	**0.98 (0.15)**	**0.97 (0.14)**			
Knee adduction moment (% BW × HT)						
First peak	0.32 (0.07)	**0.24 (0.05)**	**0.24 (0.05)**	2.30 (0.89)	**1.83 (0.74)**	**1.76 (0.75)**
Second peak	0.14 (0.03)	**0.12 (0.04)**	**0.11 (0.03)**			

Results are presented as mean (standard deviation). Two peaks are assessed during level waking, while one peak is assessed during standing up from a chair. Knee joint moments are normalised by bodyweight times height (BW $\times$ HT). Bold indicates a significant difference (*p* < 0.05) when comparing the peak values from cost functions (2) and (3) and those from the default cost function in the intact limb

**Fig. 2:**
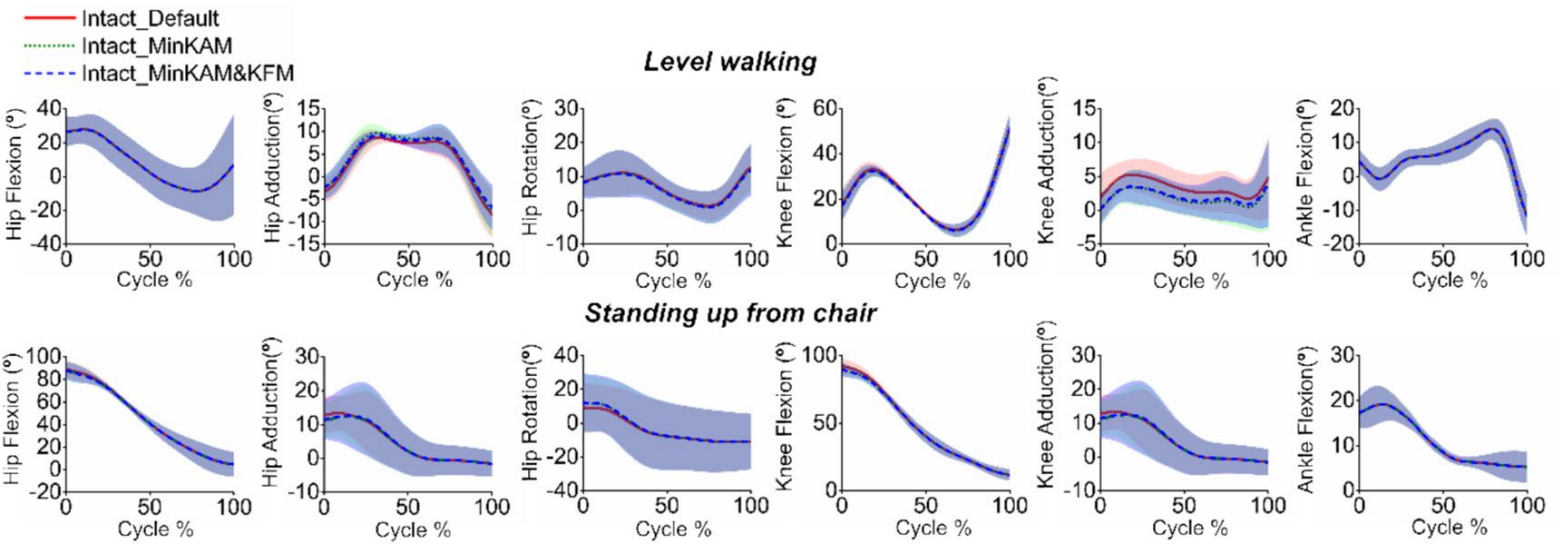
Simulated joint kinematics in the intact limb using three cost functions during level walking and standing up from a chair (mean -solid or dashed lines - ± standard deviation - shaded areas): (1) default (red solid line), (2) minimising knee adduction moment (MinKAM, green dotted line) and (3) minimising knee adduction moment and knee flexion moment (MinKAM&KFM, blue dashed line).

Both the MinKAM and MinKAM&KFM cost functions resulted in changes in joint kinematics. On the frontal plane, MinKAM and MinKAM&KFM resulted in decreased peak knee adduction angles by an average of 29% (*p* = 0.087) and 26% (*p* = 0.100), respectively; however, these reductions were not statistically significant. Additionally, the cost functions resulted in increased peak hip adduction angles in the first half of stance, by an average of 12% (*p* = 0.013) and 6% (*p* = 0.563), respectively. On the transverse plane, during the initial phase of standing up from a chair, MinKAM and MinKAM&KFM resulted in increased peak hip internal rotation angles by an average of 47% (*p* = 0.068) and 54% (*p* = 0.094), respectively (Fig. 2).

**Fig. 3:**
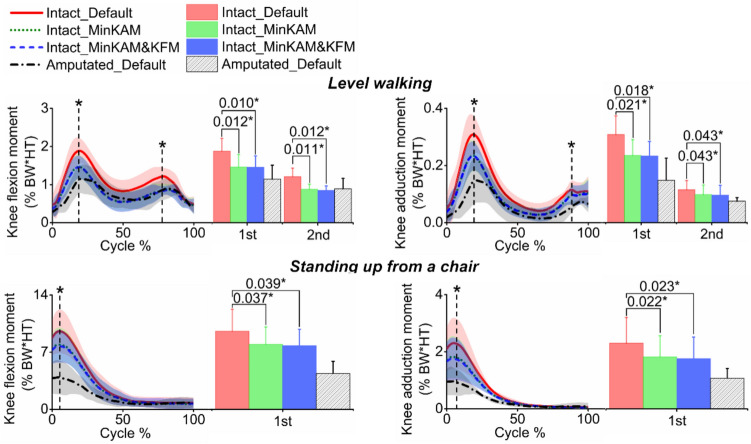
Simulated knee flexion moment (left) and knee adduction moment (right) in the intact limb using three cost functions during level walking and standing up from a chair (mean -solid or dashed lines - ± standard deviation - shaded areas): (1) default (red solid line), (2) minimising knee adduction moment (MinKAM, green dotted line) and (3) minimising knee adduction moment and knee flexion moment (MinKAM&KFM, blue dashed line). Knee adduction moment and knee flexion moment in the amputated limb (black dashed dotted line with default cost function) are also presented. Knee joint moments are normalised by bodyweight times height (BW $\times$ HT). Bars represent peak values; error bars represent standard deviations; horizontal bars denote *p*-values when comparing the predicted knee joint moment using cost functions of (2) and (3) with that using the default cost function. Bars with an asterisk (*) indicate a significant difference (*p* < 0.05).

Both the MinKAM and MinKAM&KFM cost functions reduced knee joint moments (as shown in Fig. 3; Table 1). In level walking, MinKAM and MinKAM&KFM resulted in an average reduction of 25% (*p* = 0.012) and 24% (*p* = 0.010) for the peak knee flexion moment, respectively, and an average reduction of 22% (*p* = 0.021) and 22% (*p* = 0.018) for the peak knee adduction moment, respectively. In standing up from a chair, MinKAM and MinKAM&KFM resulted in an average reduction of 25% (*p* = 0.037) and 24% (*p* = 0.039) for the peak knee flexion moment, respectively, and an average reduction of 17% (*p* = 0.022) and 19% (*p* = 0.023) for the peak knee adduction moment, respectively.

**Fig. 4:**
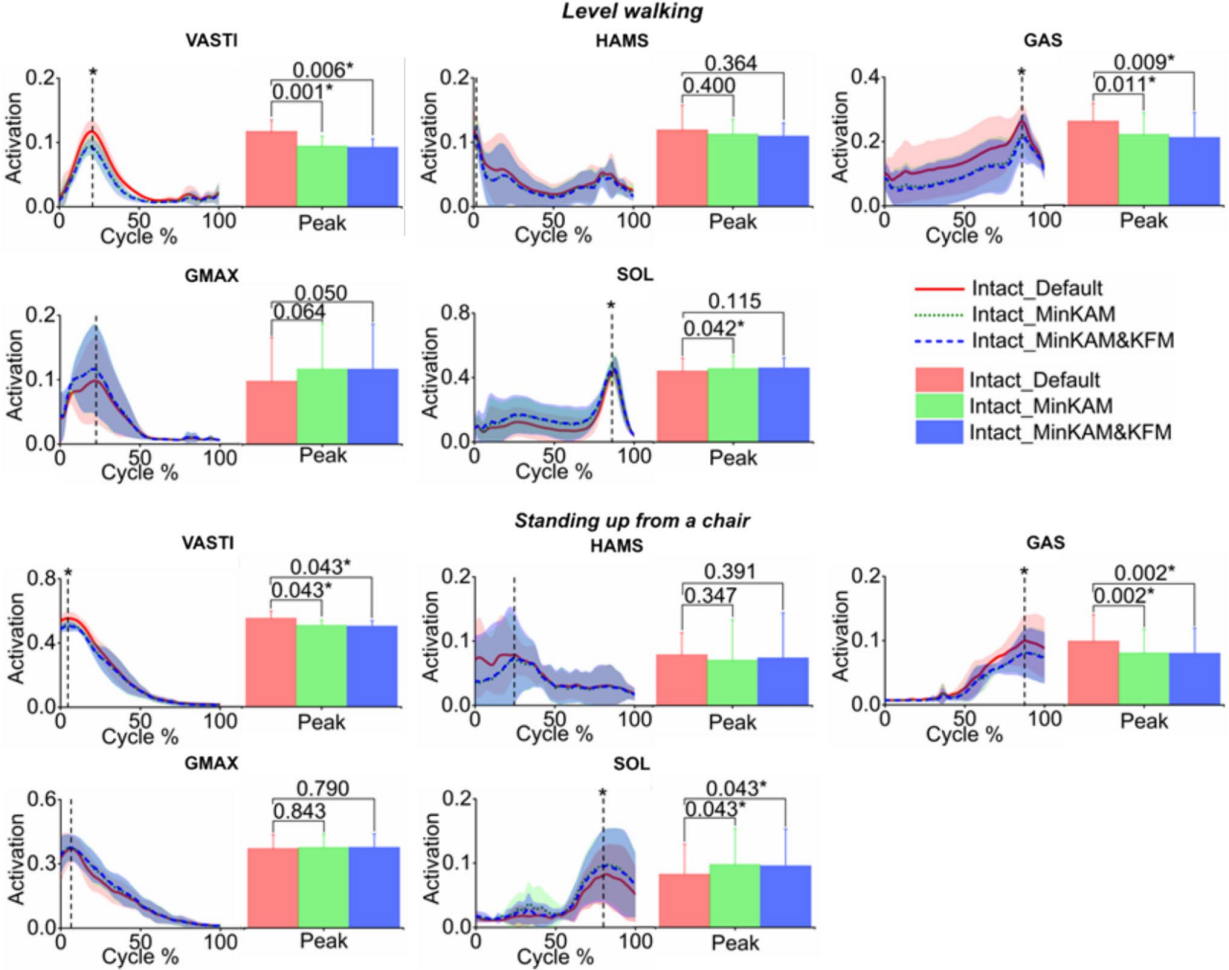
Simulated muscle activations in the intact limb using three cost functions during level walking and standing up from a chair chair (mean -solid or dashed lines - ± standard deviation - shaded areas): (1) default (red solid line), (2) minimising knee adduction moment (MinKAM, green dotted line) and (3) minimising knee adduction moment and knee flexion moment (MinKAM&KFM, blue dashed line). Bars represent peak values; error bars represent standard deviations; and horizontal bars denote *p*-values when comparing the predicted muscle activations using cost functions (2) and (3) with that using the default cost function. Asterisk (*) indicates a significant difference (*p* < 0.05). Muscle symbols are: *VASTI* vastus medialis, vastus lateralis and vastus intermedius combined; *HAMS* semimembranosus, semitendinosus and biceps femoris long head combined; *GAS* gastrocnemius, *GMAX* gluteus maximus and *SOL* soleus

Both MinKAM and MinKAM&KFM cost functions resulted in decreased muscle activations in VASTI (vastus medialis, vastus lateralis and vastus intermedius combined), HAMS (semimembranosus, semitendinosus and biceps femoris combined) and GAS (medial and lateral gastrocnemius combined). In both activities, significant reductions were found in peak activations of VASTI (*p* ≤ 0.043) and GAS (*p* ≤ 0.011). In addition, both cost functions resulted in increased muscle activation of GMAS (gluteus maximus) and SOL (soleus), with a significant increase found in the peak activation of SOL (*p* ≤ 0.043) (Fig. 4; Table 2).

**Table 2:** Simulated changes in peak muscle activations in the intact limb using minimising knee adduction moment (MinKAM) and minimising knee adduction moment and knee flexion moment (MinKAM&KFM) cost functions during level walking and standing up from a chair, compared to the default cost function

	Change in the peak muscle activation			
	Level walking		Standing up from a chair	
	MinKAM	MinKAM&KFM	MinKAM	MinKAM&KFM
VASTI	**− 0.19(0.05)**	**− 0.20(0.09)**	**− 0.07(0.05)**	**− 0.09(0.06)**
HAMS	− 0.05(0.15)	− 0.06(0.16)	− 0.23(0.41)	− 0.23(0.44)
GAS	**− 0.18(0.09)**	**− 0.21(0.13)**	**− 0.18(0.04)**	**− 0.20(0.06)**
GMAX	+ 0.14(0.04)	+ 0.14(0.04)	+ 0.01(0.05)	+ 0.06(0.06)
SOL	** + 0.06(0.06)**	** + 0.08(0.09)**	**0.35 (0.34)**	**0.17(0.10)**

Results are presented as mean (standard deviation). Bold indicates a significant difference (*p* < 0.05). Muscle symbols are: VASTI, vastus medialis, vastus lateralis and vastus intermedius combined; HAMS, semimembranosus, semitendinosus and biceps femoris long head combined; GAS, gastrocnemius; GMAX, gluteus maximus and SOL, soleus

**Fig. 5:**
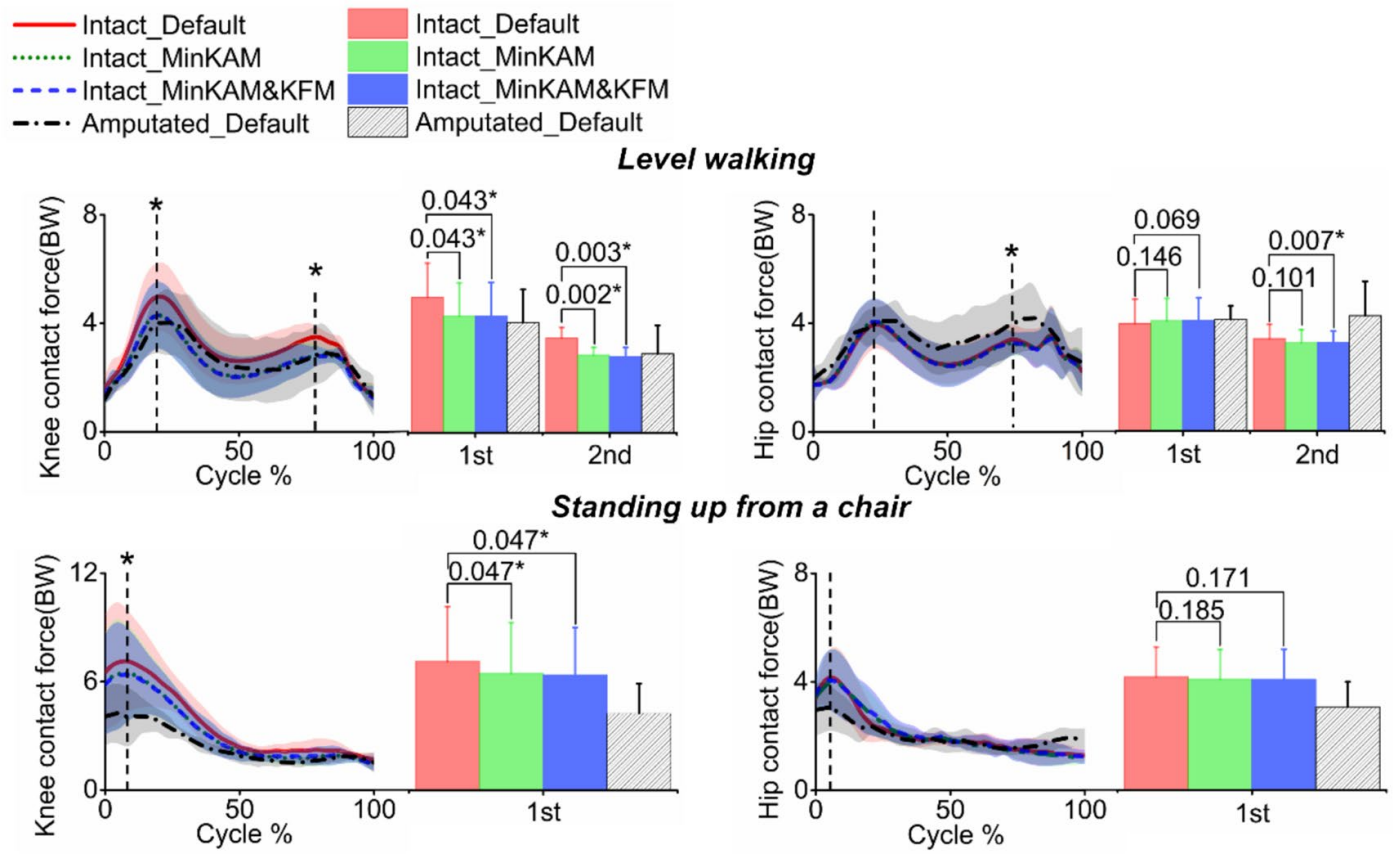
Simulated knee contact force (left) and hip contact force (right) in the intact limb using three cost functions (mean -solid or dashed lines - ± standard deviation - shaded areas): (1) default (red solid line), (2) minimising knee adduction moment (MinKAM, green dotted line) and (3) minimising knee adduction moment and knee flexion moment (MinKAM&KFM, blue dashed line) during level walking and standing up from a chair. The knee and hip contact forces in the amputated limb (black dashed dotted line, default cost function) are also presented. Joint contact forces are normalised by bodyweight (BW). Bars represent peak values; error bars represent standard deviations; horizontal bars denote *p*-values when comparing the predicted joint contact forces using cost functions of (2) and (3) with that using the default cost function. Bars with an asterisk (*) indicate a significant difference (*p* < 0.05).

Both MinKAM and MinKAM&KFM significantly decreased peaks of knee contact force (as shown in Fig. 5; Table 3). During level walking, the first and second peaks were deceased by an average of 13% (*p* = 0.043) and 16% (*p* = 0.002) using MinKAM, and by 13% (*p* = 0.043) and 17% (*p* = 0.003) using MinKAM&KFM. During standing up from a chair, both cost functions resulted in an average reduction of 13% (*p* = 0.047) and 14% (*p* = 0.047) in peaks, respectively. MinKAM&KFM also resulted in a significant reduction of 4% (*p* = 0.007) in the second peak hip contact force during level walking.

**Table 3:** Simulated peak knee and hip joint contact forces in the intact limb, using three cost functions (mean ± standard deviation): (1) default, (2) minimising knee adduction moment (MinKAM) and (3) minimising knee adduction moment and knee flexion moment (MinKAM&KFM), during both level walking and standing up from a chair

	Level walking			Standing up from a chair		
	Default	MinKAM	MinKAM&KFM	Default	MinKAM	MinKAM&KFM
Hip contact force (BW)						
First peak	3.96 (0.89)	4.06 (0.82)	4.07 (0.84)	4.16 (1.10)	4.08 (1.09)	4.07 (1.11)
Second peak	3.40 (0.53)	3.25 (0.48)	**3.26 (0.42)**			
Knee contact force (BW)						
First peak	5.04 (1.26)	**4.36 (1.19)**	**4.36 (1.21)**	**8.96 (1.99)**	**7.82 (1.53)**	**7.70 (1.45)**
Second peak	3.63 (0.45)	**3.05 (0.42)**	**3.02 (0.45)**			

Two peaks are assessed during level waking, while one peak is assessed during standing up from a chair. Joint contact forces are normalised by bodyweight (BW). Bold indicates a significant difference (*p* < 0.10) when comparing the peak values from cost functions (2) and (3) and those from the default cost function in the intact limb

**Fig. 6:**
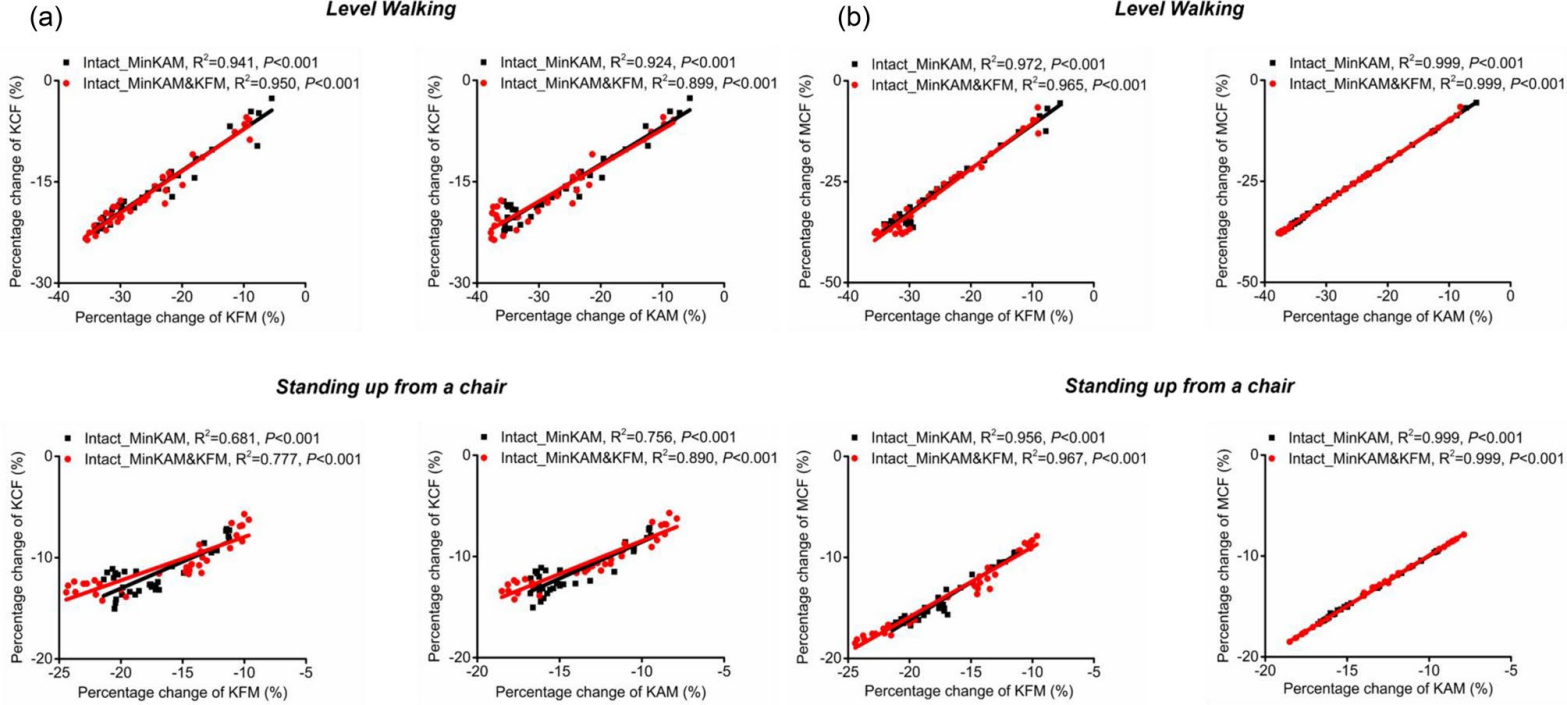
The correlation between the percentage changes of knee flexion moment (KFM) / knee adduction moment (KAM) and the percentage changes of knee contact force (KCF, a) and medial knee contact force (MCF, b) in the level walking and standing up from a chair. Simulated knee joint moments and knee contact forces are from three cost functions: (1) default, (2) minimising knee adduction moment (MinKAM) and (3) minimising knee adduction moment and knee flexion moment (MinKAM&KFM). The percentage changes are calculated by comparing the results of cost functions (2) and (3) to the result of default cost function, then diving the difference is by the result of the default cost function.

As shown in Fig. 6, during level walking, the percentage change of knee flexion moment and the percentage change of knee adduction moment both displayed good correlation with the percentage change of medial and total knee contact force (*R*^2^ ≥ 0.899). During standing up from a chair, the percentage change of knee flexion moment displayed moderate to good (*R*^2^ ≥ 0.681) correlation with the percentage change of medial and total knee contact force, while the percentage change of knee adduction moment displayed good correlation with the percentage change of medial and total knee contact force (*R*^2^ ≥ 0.756).

## Discussion

We implemented muscle-driven simulations of walking and standing up from a chair for people with unilateral transtibial amputations, in order to explore muscle coordination strategies aimed at reducing excessive joint loading at the knee of the intact limb. Results showed that to mitigate peaks at the intact knee, muscle activations of vasti and gastrocnemius should be decreased while muscle activations of soleus should be increased during activities of level walking and standing up from a chair. Additionally, a gait retraining strategy was suggested: decreasing the knee adduction angles during stance.

Gait retraining techniques (e.g., medial thrust and trunk lean) have demonstrated that reducing knee adduction angles effectively decreases knee adduction moments [14]. Our modelling study had the same conclusion. The cost function of minimising KAM resulted in reduced knee adduction angles with a maximum reduction of up to 1.7° during stance. This alternation, however, may lead to increased hip adduction angles: at the first half of stance, a maximum increase was up to 1.1°. A similar pattern was shown in the physical study. Consequently, we noticed a slight increase of the first peak of the hip joint contact force; however, the increasement was not significant (Fig.5).

The modelled reductions in knee adduction moments using the minimising KAM and KAM&KFM cost functions fall within the range reported in experimental methods (between 7 and 65%) [13, 30, 31]. Additionally, previous findings have shown that alleviating self-reported knee pain can be achieved by decreasing the first peak KAM by 20% in OA patients [32]. This indicates the practical functional implementation of our proposed muscle coordination strategies, with great potential to reduce knee joint loading and pain in people with limb loss. Notably, this mitigation of knee joint loading reduced the peaks of hip joint loading, despite the reduction not being significant.

It is worth noting that muscle coordination strategies may vary during different daily activities. When compared to level walking, a greater knee adduction moment was observed in standing up from a chair. Knee adduction moment is often used as a surrogate measure of internal medial knee contact force, and our findings suggest that reducing the knee adduction moment had greater mechanical efficacy during standing up from a chair, aiming to reduce internal medial knee contact force.

Muscle coordination strategies were identified to reduce knee contact forces. Generally, two muscles -- vasti and gastrocnemius -- should be deactivated during both level walking and standing up from a chair. The reduction of knee contact forces can be achieved by deactivating vasti in the early stage and deactivating gastrocnemius in the late stage (Fig. 4). While previous muscle coordination strategies of gastrocnemius avoidance only successfully reduced the second peak of knee contact force [21], our proposal was found successful in reducing both peaks, with the first peak considered a better indicator of the development and progression of knee OA [39, 40]. Our findings also reveal the need to activate the soleus in the late stage to assist the gastrocnemius in fulfilling the task. This increased activation of the soleus was found to not increase the knee joint force.

In level walking, minimising both the knee flexion moment and knee adduction moment proved effective in reducing knee joint forces, while in standing up from the chair, minimising the knee adduction moment was more effective, as indicated by the stronger correlation between the percentage changes of knee adduction moment and knee contact force.

There are known limitations in this study. First, we acknowledge that the small sample size in our study may limit the generalizability of the findings. A larger participant pool could have provided a more comprehensive analysis, allowing for better insights into how factors such as musculature geometry, body segment properties, and physical activity levels might influence the results. Second, the prosthesis was modelled as three identical components -- socket, stump, and foot -- with the same body segment properties, which could influence the joint moment of the residual limb [41, 42]. Thirdly, the knee joint model implemented in this study did not permit medial and lateral knee joint contact force to be quantified separately and the knee internal-external rotational degree of freedom was locked. This necessitated the use of a surrogate measure of medial knee joint contact force to be calculated. However, the conclusions have focused mainly on the overall knee joint force, which was directly calculated in this study. Finally, we did not simulate foot-ground interaction using contact spheres. Only alterations in gait kinematics and its resultant alternations in muscle activations were analysed, given the direct inputs of measured ground reaction forces. Future studies should consider alterations in ground reaction force to minimize knee joint loading.

In conclusion, this study presented simulation-based muscle coordination strategies and kinematic patterns capable of alleviating knee joint loading in the intact limb during level walking and standing up from a chair for people with unilateral transtibial amputation. Our findings indicated that both minimising KAM and KAM&KFM were effective in reducing joint loading during level walking, while minimising KAM&KFM could be more effective in reducing joint loading during standing up from a chair. Therefore, alternation of muscle coordination and kinematic patterns should be carefully designed for different daily activities.

## Supplementary Information

Below is the link to the electronic supplementary material.

Supplementary file1 (PDF 3272 kb)

## References

[1] Nowygrod, R., N. Egorova, G. Greco, P. Anderson, A. Gelijns, A. Moskowitz, J. McKinsey, N. Morrissey, and K. C. Kent. Trends, complications, and mortality in peripheral vascular surgery. *J. Vasc. Surg.* 43:205–216, 2006. 16476588 10.1016/j.jvs.2005.11.002

[2] Ahmad, N., G. N. Thomas, P. Gill, C. Chan, and F. Torella. Lower limb amputation in England: prevalence, regional variation and relationship with revascularisation, deprivation and risk factors. A retrospective review of hospital data. *J. R. Soc. Med.* 107:483–489, 2014. 25389229 10.1177/0141076814557301 PMC4265106

[3] Bateni, H., and S. J. Olney. Kinematic and kinetic variations of below-knee amputee gait. *J. Prosthet. Orthot.* 14:2–10, 2002.

[4] Sanderson, D. J., and P. E. Martin. Lower extremity kinematic and kinetic adaptations in unilateral below-knee amputees during walking. *Gait Posture.* 6:126–136, 1997.

[5] van der Kruk, E., A. K. Silverman, L. Koizia, P. Reilly, M. Fertleman, and A. M. Bull. Age-related compensation: neuromusculoskeletal capacity, reserve & movement objectives. *J. Biomech.*122:110385, 2021. 33910081 10.1016/j.jbiomech.2021.110385

[6] Aruin, A., J. Nicholas, and M. L. Latash. Anticipatory postural adjustments during standing in below-the-knee amputees. *Clin. Biomech.* 12:52–59, 1997. 10.1016/s0268-0033(96)00053-8 11415672

[7] Isakov, E., H. Burger, J. Krajnik, M. Gregoric, and C. Marincek. Knee muscle activity during ambulation of trans-tibial amputees. *J. Rehabil. Med.* 33:196–199, 2001. 11585149 10.1080/165019701750419572

[8] Ding, Z., D. P. Henson, B. Sivapuratharasu, A. H. McGregor, and A. M. Bull. The effect of muscle atrophy in people with unilateral transtibial amputation for three activities: gait alone does not tell the whole story. *J. Biomech.*149:111484, 2023. 36791515 10.1016/j.jbiomech.2023.111484

[9] Devan, H., A. Carman, P. Hendrick, L. Hale, and D. C. Ribeiro. Spinal, pelvic, and hip movement asymmetries in people with lower-limb amputation: systematic review. *J. Rehabil. Res. Dev.* 52:1–20, 2015. 26186283 10.1682/JRRD.2014.05.0135

[10] Gailey, R., K. Allen, J. Castles, J. Kucharick, and M. Roeder. Review of secondary physical conditions associated with lower-limb. *J. Rehabil. Res. Dev.* 45:15–30, 2008. 18566923 10.1682/jrrd.2006.11.0147

[11] Norvell, D. C., J. M. Czerniecki, G. E. Reiber, C. Maynard, J. A. Pecoraro, and N. S. Weiss. The prevalence of knee pain and symptomatic knee osteoarthritis among veteran traumatic amputees and nonamputees. *Arch. Phys. Med. Rehabil.* 86:487–493, 2005. 15759233 10.1016/j.apmr.2004.04.034 PMC11803826

[12] Simic, M., T. V. Wrigley, R. S. Hinman, M. A. Hunt, and K. L. Bennell. Altering foot progression angle in people with medial knee osteoarthritis: the effects of varying toe-in and toe-out angles are mediated by pain and malalignment. *Osteoarthr. Cartil.* 21:1272–1280, 2013. 10.1016/j.joca.2013.06.001 23973141

[13] Shull, P. B., R. Shultz, A. Silder, J. L. Dragoo, T. F. Besier, M. R. Cutkosky, and S. L. Delp. Toe-in gait reduces the first peak knee adduction moment in patients with medial compartment knee osteoarthritis. *J. Biomech.* 46:122–128, 2013. 23146322 10.1016/j.jbiomech.2012.10.019

[14] Gerbrands, T. A., M. F. Pisters, P. J. R. Theeven, S. Verschueren, and B. Vanwanseele. Lateral trunk lean and medializing the knee as gait strategies for knee osteoarthritis. *Gait Posture.* 51:247–253, 2016. 27838568 10.1016/j.gaitpost.2016.11.014

[15] Walter, J. P., D. D. D’Lima, C. W. Colwell Jr., and B. J. Fregly. Decreased knee adduction moment does not guarantee decreased medial contact force during gait. *J. Orthop. Res.* 28:1348–1354, 2010. 20839320 10.1002/jor.21142 PMC2984615

[16] Birmingham, T. B., M. A. Hunt, I. C. Jones, T. R. Jenkyn, and J. R. Giffin. Test-retest reliability of the peak knee adduction moment during walking in patients with medial compartment knee osteoarthritis. *Arthritis. Care. Res.* 57:1012–1017, 2007. 10.1002/art.22899 17665490

[17] Hurwitz, D., A. Ryals, J. Case, J. Block, and T. Andriacchi. The knee adduction moment during gait in subjects with knee osteoarthritis is more closely correlated with static alignment than radiographic disease severity, toe out angle and pain. *J. Orthop. Res.* 20:101–107, 2002. 11853076 10.1016/S0736-0266(01)00081-X

[18] Sharma, L., D. E. Hurwitz, E. J. Thonar, J. A. Sum, M. E. Lenz, D. D. Dunlop, T. J. Schnitzer, G. Kirwanmellis, and T. P. Andriacchi. Knee adduction moment, serum hyaluronan level, and disease severity in medial tibiofemoral osteoarthritis. *Arthritis Rheumatol.* 41:1233–1240, 1988. 10.1002/1529-0131(199807)41:7<1233::AID-ART14>3.0.CO;2-L 9663481

[19] Kutzner, I., A. Trepczynski, M. O. Heller, and G. Bergmann. Knee adduction moment and medial contact force–facts about their correlation during gait. *PLoS ONE*.8:e81036, 2013. 24312522 10.1371/journal.pone.0081036 PMC3847086

[20] Manal, K., E. Gardinier, T. S. Buchanan, and L. Snyder-Mackler. A more informed evaluation of medial compartment loading: the combined use of the knee adduction and flexor moments. *Osteoarthr. Cartil.* 23:1107–1111, 2015. 10.1016/j.joca.2015.02.779 PMC4470852 25862486

[21] Uhlrich, S. D., R. W. Jackson, A. Seth, J. A. Kolesar, and S. L. Delp. Muscle coordination retraining inspired by musculoskeletal simulations reduces knee contact force. *Sci. Rep.* 12:9842, 2022. 35798755 10.1038/s41598-022-13386-9 PMC9262899

[22] Rane, L., and A. M. J. Bull. Functional electrical stimulation of gluteus medius reduces the medial joint reaction force of the knee during level walking. *Arthritis. Res. Ther.* 18:1–11, 2016. 27809923 10.1186/s13075-016-1155-2 PMC5094077

[23] DeMers, M. S., S. Pal, and S. L. Delp. Changes in tibiofemoral forces due to variations in muscle activity during walking. *J. Orthop. Res.* 32:769–776, 2014. 24615885 10.1002/jor.22601 PMC4409006

[24] Miller, R. H., S. C. Brandon, and K. J. Deluzio. Predicting sagittal plane biomechanics that minimize the axial knee joint contact force during walking. *J. Biomech. Eng.* 135:011007, 2013. 23363218 10.1115/1.4023151

[25] van Veen, B., E. Montefiori, L. Modenese, C. Mazzà, and M. Viceconti. Muscle recruitment strategies can reduce joint loading during level walking. *J. Biomech.*97:109368, 2019. 31606129 10.1016/j.jbiomech.2019.109368

[26] Azmi, N. L., Z. Ding, R. Xu, and A. M. Bull. Activation of biceps femoris long head reduces tibiofemoral anterior shear force and tibial internal rotation torque in healthy subjects. *PLoS ONE*.13:e0190672, 2018. 29304102 10.1371/journal.pone.0190672 PMC5755889

[27] Willson, A. M. (2017) A quasi-passive biarticular prosthesis and novel musculoskeletal model for transtibial amputees.

[28] Meireles, S., F. De Groote, N. D. Reeves, S. Verschueren, C. Maganaris, F. Luyten, and I. Jonkers. Knee contact forces are not altered in early knee osteoarthritis. *Gait Posture*. 45:115–120, 2016. 26979892 10.1016/j.gaitpost.2016.01.016

[29] Rajagopal, A., C. L. Dembia, M. S. DeMers, D. D. Delp, J. L. Hicks, and S. L. Delp. Full-body musculoskeletal model for muscle-driven simulation of human gait. *IEEE Trans. Biomed. Eng.* 63:2068–2079, 2016. 27392337 10.1109/TBME.2016.2586891 PMC5507211

[30] Millard, M., T. Uchida, A. Seth, and S. L. Delp. Flexing computational muscle: modeling and simulation of musculotendon dynamics. *J. Biomech. Eng.*135:021005, 2013. 23445050 10.1115/1.4023390 PMC3705831

[31] Koelewijn, A. D., and A. J. van den Bogert. Joint contact forces can be reduced by improving joint moment symmetry in below-knee amputee gait simulations. *Gait Posture.* 49:219–225, 2016. 27459416 10.1016/j.gaitpost.2016.07.007

[32] LaPrè, A., M. Price, R. Wedge, B. Umberger, and F. C. Sup IV. Approach for gait analysis in persons with limb loss including residuum and prosthesis socket dynamics. *Int. J. Numer. Method Biomed. Eng.*34:e2936, 2018. 29111608 10.1002/cnm.2936

[33] Delp, S. L., F. C. Anderson, A. S. Arnold, P. Loan, A. Habib, et al. OpenSim: open-source software to create and analyze dynamic simulations of movement. *IEEE Trans. Biomed. Eng.* 54:1940–1950, 2007. 18018689 10.1109/TBME.2007.901024

[34] Dembia, C. L., N. A. Bianco, A. Falisse, J. L. Hicks, and S. L. Delp. Opensim moco: musculoskeletal optimal control. *PLoS Comput. Biol.*16:e1008493, 2020. 33370252 10.1371/journal.pcbi.1008493 PMC7793308

[35] Todorov, E., Li, W. Optimal control methods suitable for biomechanical systems. In: Proceedings of the 25th Annual International Conference of the IEEE Engineering in Medicine and Biology Society (IEEE Cat. No. 03CH37439). IEEE 2:1758–1761, 2003 10.1109/IEMBS.2004.1404280 17271336

[36] Park, E. S., C.-I. Park, H. J. Lee, D. Y. Kim, D. S. Lee, and S.-R. Cho. The characteristics of sit-to-stand transfer in young children with spastic cerebral palsy based on kinematic and kinetic data. *Gait Posture.* 17:43–49, 2003. 12535725 10.1016/s0966-6362(02)00055-3

[37] Lin, Y.-T., and H.-J. Lee. Comparison of the lower extremity kinematics and center of mass variations in sit-to-stand and stand-to-sit movements of older fallers and nonfallers. *Arch. Rehabil. Res. Clin. Transl.*4:100181, 2022. 35243318 10.1016/j.arrct.2022.100181 PMC8867046

[38] Trepczynski, A., I. Kutzner, G. Bergmann, W. R. Taylor, and M. O. Heller. Modulation of the relationship between external knee adduction moments and medial joint contact forces across subjects and activities. *Arthritis Rheumatol.* 66:1218–1227, 2014. 24470261 10.1002/art.38374 PMC4158863

[39] Mündermann, A., C. O. Dyrby, and T. P. Andriacchi. Secondary gait changes in patients with medial compartment knee osteoarthritis: increased load at the ankle, knee, and hip during walking. *Arthritis Rheumatol.* 52:2835–2844, 2005. 10.1002/art.21262 16145666

[40] Baliunas, A., D. Hurwitz, A. Ryals, A. Karrar, J. Case, J. Block, and T. Andriacchi. Increased knee joint loads during walking are present in subjects with knee osteoarthritis. *Osteoarthr. Cartil.* 10:573–579, 2002. 10.1053/joca.2002.0797 12127838

[41] Czerniecki, J. M., A. Gitter, and C. Munro. Joint moment and muscle power output characteristics of below knee amputees during running: the influence of energy storing prosthetic feet. *J. Biomech.* 24:63–75, 1991. 2026634 10.1016/0021-9290(91)90327-j

[42] Fey, N. P., G. K. Klute, and R. R. Neptune. Altering prosthetic foot stiffness influences foot and muscle function during below-knee amputee walking: a modeling and simulation analysis. *J. Biomech.* 46:637–644, 2013. 23312827 10.1016/j.jbiomech.2012.11.051

