# Supplementary material for: Alterations in Muscle Coordination to Reduce Knee Joint Loading for People with Limb Loss
Source: Ann Biomed Eng. 2025 Jan 24;53(4):935–45. doi: 10.1007/s10439-025-03682-6 (PMC11929717; doi:10.1007/s10439-025-03682-6)

Supplementary file

Table S1. The root-mean-square-error (RMSE) and the square of Pearson correlation coefficient (R^2) between predicted joint kinematics using the default cost function and the experimental data. Results are averaged across all subjects.

	Level walking				Standing up from a chair			
	Intact limb		Amputated limb		Intact limb		Amputated limb	
	RSME (°) Mean±std	R^2	RSME (°) Mean±std	R^2	RSME (°) Mean±std	R^2	RSME (°) Mean±std	R^2
Hip Flexion	3.09±1.23	0.992	3.80±1.22	0.997	3.08±0.57	0.996	2.73±1.13	0.999
Hip Adduction	2.53±1.48	0.958	2.34±1.10	0.848	3.04±0.94	0.870	3.91±1.40	0.697
Hip Rotation	1.98±2.17	0.962	1.32±0.61	0.947	2.66±1.50	0.912	1.39±0.88	0.983
Knee Flexion	1.45±0.72	0.988	2.16±0.43	0.946	1.88±1.11	0.997	2.82±0.96	0.994
Kne Adduction	2.99±2.46	0.642	1.88±0.93	0.640	4.70±1.26	0.777	1.72±0.77	0.901
Ankle plantarflexion	0.71±0.48	0.998	1.14±0.43	0.998	0.70±0.51	0.988	0.19±0.09	0.996

Fig. S1 Predicted joint kinematics using the default cost function (dashed dotted line) compared to experimental data across all participants.

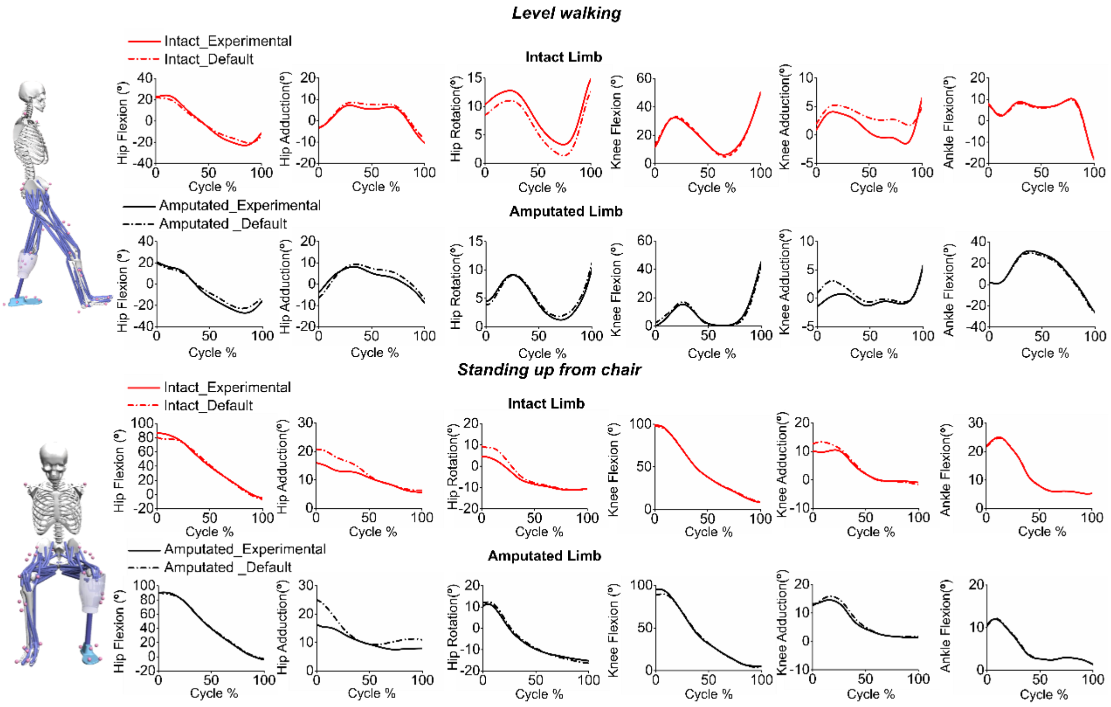

Supplement: Supplementary file 1 — Supplementary file1 (PDF 3272 kb) [file 10439_2025_3682_MOESM1_ESM.pdf]
